# Facilitation of arthroscopic visualization and treatment of meniscal tears using a stifle joint distractor in the dog

**DOI:** 10.1186/s12917-018-1534-9

**Published:** 2018-06-28

**Authors:** Gian Luca Rovesti, Veronica Devesa, Laura Bertorelli, Jesus Rodriguez-Quiros

**Affiliations:** 1 Clinica Veterinaria M. E. Miller, Cavriago, Italy; 2 Madrid, Spain; 3 Department of Internal Medicine and Surgery, Veterinary Teaching Hospital, Veterinary School, Complutense University, Madrid, Spain

**Keywords:** Arthroscopy, Stifle, Joint distraction, Meniscal surgery, Dog

## Abstract

**Background:**

Stifle arthroscopy has been described to have high sensitivity and specificity in the evaluation of menisci in dogs, particularly for the medial meniscus. However, the visualization of menisci can be difficult. The use of femoral distractors in human medicine has been described to simplify demanding surgical procedures, such as meniscus transplantation. In veterinary medicine, stifle distraction has been reported to facilitate access to the joint and visualization of intra-articular structures, but there are no studies reporting the use of a stifle distraction technique while performing challenging surgical procedures, such as meniscal suture, in clinical patients.

The objective of this study was to evaluate the feasibility, effectiveness and safety of stifle distraction to achieve consistent visualization of menisci and to facilitate performing arthroscopic procedures in clinical patients with stifle disease. Initial arthroscopic evaluation of the stifle joint was performed without distraction in the study population consisting of 13 dogs with naturally occurring stifle disease. The criteria for inclusion was prospectively set as the observation of a frank disease or anomaly of the menisci that could not be further treated or clarified without the risk of damaging the joint cartilage due to the requested manoeuvres. After the first examination, distraction was applied in order to complete the assessment of menisci. After achieving an accurate diagnosis, partial meniscectomy or meniscal repair was performed as needed while maintaining the distraction.

**Results:**

Complete visualization and assessment of menisci were achieved thanks to the use of distraction. This manoeuvre facilitated access to the required area of the involved meniscus, and meniscal treatment could be successfully performed without damaging the articular cartilage. During the follow-up period, no postoperative complication related to the distraction or to the arthroscopic procedure was observed.

**Conclusions:**

Stifle joint distraction during arthroscopy in dogs improves visualization of both menisci, and particularly the caudal horn of the medial meniscus. Despite being a subjective assessment, it is the authors’ opinion that this procedure also increases the ease of performing challenging procedures like meniscal suture, as it enlarges the space available to reach the correct working angulations.

## Background

Stifle arthroscopy has been reported to be the most accurate diagnostic method for evaluating meniscal pathology and for carrying out its treatment, while arthrotomy has resulted in a higher incidence of missed diagnosis [1, 2]. Complete meniscal evaluation in the dog may be a challenging procedure, because adequate and complete visualization of the menisci can be difficult due to the narrow joint space. In addition, in chronic cases with severe synovitis, periarticular fibrosis and capsule thickening, external manipulation may be insufficient to facilitate access to the medial compartment, and visualization of the medial meniscus is difficult [3].

Furthermore, when conditions for treatment of meniscal lesions are present, it is mandatory to have enough room available to perform the requested procedure, particularly for partial meniscectomy and meniscal suture. The latter procedure, though quite demanding, has been described [4, 5], and the potential for developing the technique could be better if the risk of iatrogenic lesions is low.

Among the techniques used to improve the visualization of the joint compartments, the use of a motorized shaver for fat pad resection and electro cautery for bleeding control have been suggested for stifle arthroscopy [6]. Infrapatellar fat pad resection can cause intra-articular haemorrhage intraoperatively, which can complicate the procedure. Furthermore, the preservation of the infrapatellar fat pad has been associated with an improved outcome in a recent study [7].

Stifle joint distraction causes an increase in the joint space, making arthroscopy easier [8, 9] and reducing the need for fat pad debridement [10]. The use of a femoral distractor has been described to facilitate meniscal treatment in human medicine [11, 12]. It has been reported to be necessary during meniscal transplantation since it facilitates suture placement [12]. In veterinary medicine, a few studies have been published regarding the use of a joint distractor in the stifle joint during arthroscopy [3, 9, 10, 13]. Kim et al. [8] described the use of a stifle distractor in cadaveric joints of toy breed dogs for performing medial meniscal release; Böttcher et al. [3] described the use of a stifle distractor technique in a case series to improve visualization of the medial meniscus and to perform partial meniscectomy if needed; Gemmil and Farrell [10] evaluated a stifle distraction technique in cadaveric stifles and client-owned dogs and performed partial meniscectomy in five cases. Finally, Winkels et al. [9] have recently described the use of the Leipzig stifle distractor to evaluate the medial meniscus in large breed dogs. However, there are no studies reporting the use of a stifle joint distraction technique while performing challenging surgical procedures such as meniscal suture in clinical patients.

Joint distraction procedures have been described as causing potential complications in human medicine, such as soft tissue injuries, fractures or neurovascular damage [14, 15], so they need to be applied carefully. In veterinary medicine, intra-articular pin placement while using an invasive pin distractor in the stifle of two dogs has been described [3]. However, other studies report no complications, tissue damage or fractures when using an invasive joint distractor in different cadaveric joints [13, 16–18]. Complications were also not observed in a recent clinical study [9].

The aim of this study was to evaluate feasibility, effectiveness and safety of the use of a joint distractor in clinical patients, in order to perform arthroscopic meniscal visualization and treatment in a predictable way, reducing the risk of iatrogenic cartilage damage.

## Methods

### Study population

Dogs were presented to the Clinica Veterinaria M.E. Miller for evaluation for hind limb lameness of at least one month duration due to stifle disease. All patients that underwent stifle arthroscopy from March 26th, 2012 to June 28th, 2013 were examined without distraction as the standard examination. All owners signed the informed consent for stifle arthroscopic examination and potential meniscal treatment. When the menisci could not be seen properly or required treatment, the distractor was used to achieve sufficient intra-articular room either to perform the requested procedure or for further examination. Thirteen stifles in client-owned dogs undergoing stifle arthroscopy for spontaneously occurring stifle disease satisfied the criteria for inclusion.

### Perioperative evaluation

Clinical and radiographic evaluations were performed, and stifle disease was diagnosed. All dogs were initially treated by the referring veterinarian with a non-steroidal anti-inflammatory drug, with no or limited improvement. The following clinical tests were performed to assess initial stifle stability: cranial drawer, tibial thrust, and varus/valgus stress. Some dogs showed no clinical stifle instability, though the clinical evaluation showed changes typical of chronic joint involvement. A procedure comprising radiographic study, arthroscopy evaluation and a potential surgical joint stabilization was scheduled in order to obtain either a final diagnosis or as part of a more complex treatment. Before the procedure, a preoperative physical examination and blood test were performed, and no concurrent abnormalities were found.

### Distraction technique

Dogs were positioned in dorsal recumbency so that the pelvic limb to be examined could be maintained in its sagittal axis by an assistant. A dog’s position was maintained with the use of a foam cushion. The unaffected hind limb was secured in the abducted position.

**Fig. 1:**
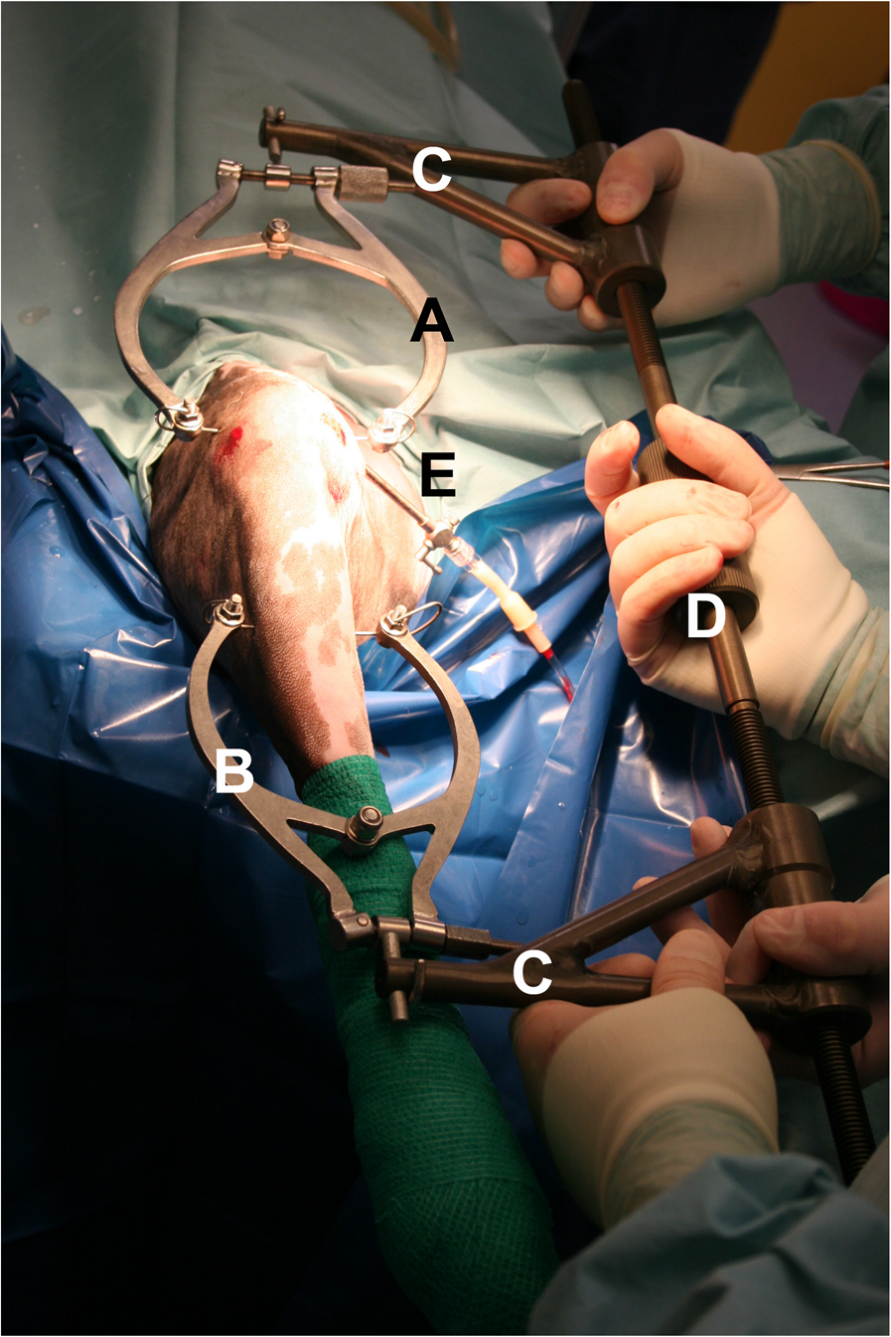
Intraoperative picture of the applied stifle distractor. The proximal stirrup (**a**) was applied in the area of the femoral condyles, while the distal stirrup (**b**) was applied in the area of the proximal tibial metaphysis. They were both connected to the bone by means of a 1.5-mm K-wire. The stirrups were then connected to the distractor’s arms (**c**), and the distraction load was increased as required by turning the knob (**d**) to achieve consistent visualization to perform a complete evaluation of the menisci and to perform the planned surgical procedure. An efflux cannula (**e**) was inserted in the proximo-medial area of the joint

The distractor device was applied as described previously [13]. A 1.5-mm diameter K-wire was placed through the femoral condyles, halfway between the patella and the fibular head in a latero-medial direction and connected to a traction stirrup (Ad Maiora s.r.l., Cavriago, Italy). A second K-wire was inserted into the middle third of the tibial diaphisis, in a medio-lateral direction, to avoid any potential interference of the hole left by the K-wire when a stabilization procedure of the stifle involving the proximal metaphyseal area was necessary. This K-wire was connected with a second traction stirrup. Then, the distractor (Titan distractor, Ad Maiora s.r.l., Cavriago, Italy) was connected to the stirrups (Fig. 1) [13].

By rotating the distraction knob to lengthen the distractor, a light distraction was applied so as to allow easy entrance into the joint with the scope and instruments. During the procedure, the distraction was increased to achieve a visualization good enough to be able to examine the complete meniscus to achieve an accurate diagnosis and perform the manoeuvres needed in each patient.

### Arthroscopic procedure

For the first examination without distraction, the aim was to visualize the proximal pouch, the patella, the femoral trochlea, femoral condyles, the cranial and caudal cruciate ligaments and both menisci, with a technique previously described [6]. After distraction was applied, the scope was introduced through a cranio-medial portal, and the palpation hook was introduced through a cranio-lateral portal (Fig. 1).

A 2.7 mm 30° fore-oblique scope was used to examine the stifle joint in medium and large breed dogs. In smaller dogs, a 2.4 mm 30° fore-oblique scope was used.

For each of the previous structures, a report of the findings was recorded.

Cartilage lesions were classified following a modified Outerbridge classification [19], while meniscal lesions were classified following the classification by Beale [6] (Table 1).

**Table 1:** Beale’s classification for meniscal lesions

a) Vertical longitudinal tears (bucket handle tears and variations)	
b) Oblique or flap tears, single or double	
c) Radial or transverse tears	
d) Horizontal tears	
e) Complex, degenerative or macerated tears	

**Fig. 2:**
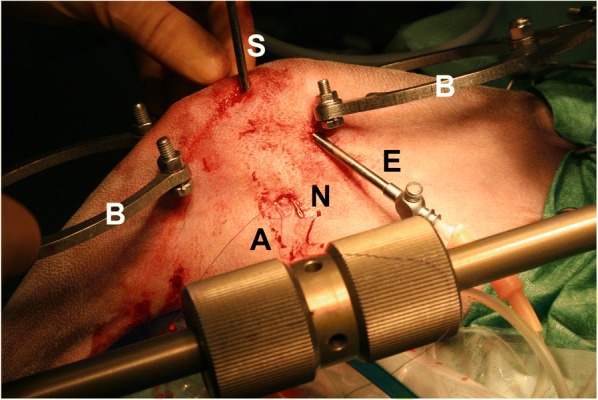
Image showing the meniscal suture procedure while distracting the stifle joint. Note that the scope (**s**) was in the cranio-medial portal, the efflux cannula (**e**) was in the proximo-medial area and the 22-G spinal needle (**n**) was retrieved in the caudo-medial area of the stifle joint through a stab wound and with minimal soft tissue dissection. The polypropylene suture extremities (**a**) were retrieved through the needle in order to perform the meniscal suture. The distraction stirrups (**b**) did not interfere with the procedure

**Fig. 3:**
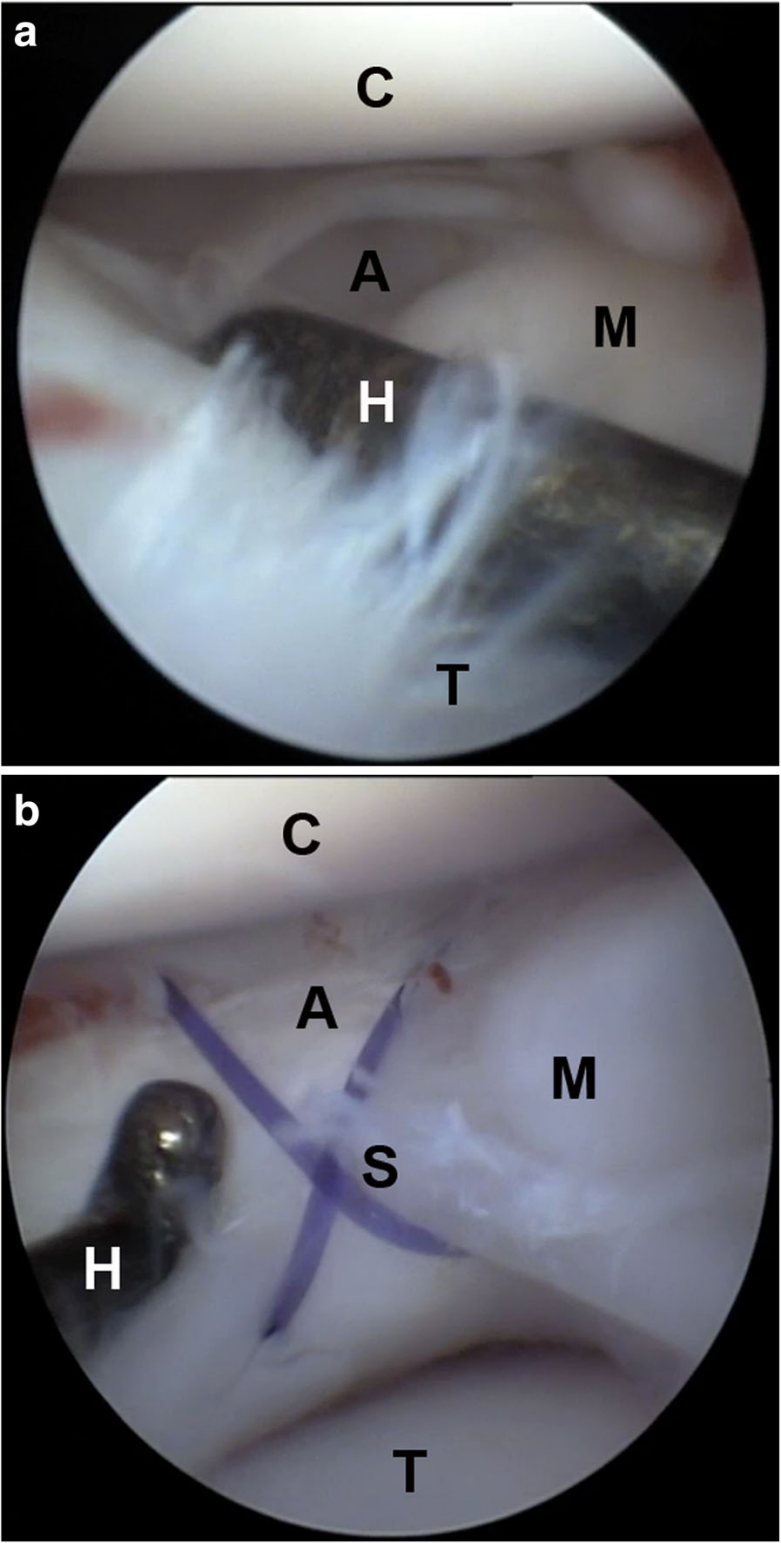
Arthroscopic images showing a bucket handle lesion (**a**) of the caudal horn of the medial meniscus (**m**) before (**a**) and after (**b**) the out-in-out meniscal suture technique (**s**) in a cruciate pattern. The joint space was increased by means of the stifle distraction device. Note the change of ratio between the joint room and the palpation hook (**h**) and the distance from the femoral condyle (**c**) and the tibial plateau (**t**) before (**a**) and after (**b**) the application of the distraction

Meniscal treatment was performed accordingly with the diagnosed lesion and it included partial meniscectomy and/or meniscal suture. Partial meniscectomy was performed in cases where meniscal tissue was damaged and displaced and when the lesion was located in the two axial thirds of the meniscus [5, 20, 21]. Meniscal suture was performed in meniscal tears located in the abaxial third of the meniscus (red zone) when enough healthy tissue was present to have sufficient holding force for the suture. The suture of the caudal horn of the medial meniscus was performed following a modified out-in-out technique already described [4, 5]. A 22-G spinal needle was inserted through the cranio-lateral portal and was directed through the meniscal area axial to the lesion. It was then pushed so that it exited in the caudo-medial area of the stifle, where it could be palpated so that its tip was exposed through a stab wound by dissecting delicate soft tissues (Fig. 2). The mandrel of the needle was removed, and a 2-metric polypropylene suture was inserted through the tip of the needle and pushed until it exited the needle hub. The suture was then locked in the hub by a finger, and the needle was retracted until the suture was brought into the joint. Under arthroscopic guidance, the needle was redirected to the desired abaxial location and inserted again through the meniscus, bringing the suture out of the tip. Once the needle tip exited again in the previous caudo-medial area, the loop of the suture was retrieved from the needle tip, leaving the two extremities of the suture exiting from the wound, and the needle was removed from the joint. Once the two extremities of the suture were available through the caudo-medial area of the stifle, a stitch was carefully tightened to lie securely on the subcutaneous fascia. The procedure was repeated as many times as needed (Fig. 3).

Clinical information and time to complete the whole surgical procedure was recorded in each case, as well as the complications encountered in performing the meniscal treatment and in visualizing the intra-articular structures.

### Postsurgical care

After surgery, pain management was performed by administration of buprenorphine (Buprenodale®, Dechra Limited, Stoke-on-Trent, United Kingdom; 0.01 mg/kg). The dogs were discharged as soon as they recovered from general anaesthesia and vital parameters were stable. They were given meloxicam (Meloxoral®, 0.1 mg/kg once daily, PO, A.T.I. s.r.l. Ozzano Emilia, Italy) for two weeks and tramadol (Altadol®, Formevet s.r.l., Milano, Italy; 3 mg/kg) for five days. Antibiotic therapy with cephalexin (ICF Vet®, I.C.F. Industria Chimica Fine s.r.l. Pignano, Italy; 20 mg/kg twice daily) was administered for one week.

### Postsurgical evaluation

Radiographic evaluation was performed after the surgical procedure in all cases. The dogs were usually rechecked clinically and radiographically at 10 and 45 days, or up to bone healing in case an osteotomy technique was performed. In addition, all dogs were clinically evaluated for up to 6 months, and radiographic examination was repeated during this time at the surgeon’s decision.

## Results

The mean bodyweight of the dogs included in the study was 29 ± 10.4 kg (range, 9.5–53 kg) and the mean age was 5.8 ± 2.8 years old (range, 1–10 years). Six dogs were males and seven females. Four were left and nine were right stifle joints.

After joint distraction, the visualization and careful assessment of the involved structures or the arthroscopic treatment of the diagnosed tear were performed.

### Cartilage and meniscal lesion classification

Cartilage and meniscal lesions were classified as described above.

A focal cartilage lesion was observed in the medial femoral condyle in one case (case 9), which was classified as grade IV. The corresponding meniscus presented radial lesions that were curetted using a tissue ablator.

Meniscal lesions were also encountered in the remaining cases (*n* = 12). A bucket handle tear was present in seven dogs (lesion type a). The lesion was close to the caudal capsule in four dogs, thus a meniscal suture was performed. The remaining three dogs had a partial meniscectomy, since the location of the lesion was in the two axial thirds and not considered adequate for meniscal healing. Two dogs had meniscal flaps (lesion type b), which were treated by partial meniscectomy in one and meniscal suture in the other. Radial meniscal tears (lesion type c) were observed on both menisci in one dog, and this was treated by radiofrequency ablation of the affected area. Two dogs had complex lesions (lesion type e) of the caudal horn of the medial meniscus that were left untreated, since degenerative signs and macerated tissue were already present (Tables 1 and 2).

**Table 2:** Population data, clinical information, treatment and time to complete the whole surgical procedure

Case	Breed	Age (y)	Weight (kg)	Lesion found and meniscal procedure	Time (minutes)
1	Rottweiler	4	53	Complex lesion caudal horn of the medial meniscus. Not treated.	30
2	Labrador Retriever	3	39	Complex lesion and color change of the medial meniscus. Not treated.	100
3	Mixed	6	27	Radial lesions in both menisci. Focal radiofrequency ablation.	45
4	Labrador Retriever	3	30	Bucket handle caudal horn medial meniscus. Meniscal suture.	90
5	Mixed	8	22	Bucket handle caudal horn medial meniscus. Meniscal suture.	100
6	Golden Retriever	1	23	Bucket handle caudal horn medial meniscus. Meniscal suture.	120
7	Mixed	3	17	Bucket handle caudal horn medial meniscus. Meniscal suture.	140
8	Labrador Retriever	8	34	Flap lesion medial meniscus. Meniscal suture.	140
9	German Shepherd	8	32	Cartilage lesione grade IV medial femoral condyle and radial lesion medial meniscus. Partial meniscectomy.	85
10	Springer Spaniel	9	24	Axial bucket handle lesion medial meniscus. Partial meniscectomy.	95
11	Jack Russel Terrier	4	9,5	Axial bucket handle lesion medial meniscus. Partial meniscectomy.	120
12	German Shepherd	9	30	Axial bucket handle lesion medial meniscus. Partial meniscectomy.	140
13	German Shepherd	10	37	Flap lesion medial meniscus. Partial meniscectomy.	145

### Treatment procedures

After the initial joint exploration, the distraction device was applied in order to complete the joint evaluation and perform the planned treatment. Its application took 11 min on average (range, 7–13 min, SD ± 1.9).

In three dogs, joint exploration revealed complex meniscal lesions or cartilage lesions that could not be repaired. In one of them, a focal radiofrequency ablation of the meniscal diseased tissue was performed (Table 2). In these dogs, the mean time to accomplish the entire procedure since the initial visualization without distraction was 58 min (range, 30–100 min; SD ± 30). The remaining dogs (*n* = 10) were diagnosed with a medial meniscal tear. Either partial meniscectomy or meniscal suture was performed according to the meniscal tissue condition. Partial meniscectomy of the medial meniscus was successfully performed in five stifles, while a meniscal suture was also successfully performed in five dogs. The mean time to complete the initial evaluation and the appropriate treatment technique was 117 ± 22 min (range, 85–145 min) (Table 2).

### Surgical and postoperative complications

The only complication encountered during the procedures was the breakage of the suture while pushing it through the meniscus by means of a needle in one dog. The reason for this was attributed to calcification of the meniscal tissue, because it felt like a slight crepitus during needle insertion. The problem was managed by repeating the procedure until it was possible to achieve the suture passing through the meniscus. No complications related to the distraction manoeuvre were observed during the surgical procedures. The manipulation of the stifle joints after arthroscopy did not reveal soft tissue damage or instability caused by the distraction procedure in any case.

During the follow-up time, no postoperative complications related to the arthroscopic or distraction procedure were observed.

## Discussion

Stifle arthroscopy has been described to have a high sensitivity and specificity in the evaluation of menisci in the dog, particularly for the medial meniscus [1]. However, the visualization of menisci can be difficult: stable joints or chronic cases may affect their evaluation. The use of a femoral distractor during arthroscopic procedures has been described in both human [11, 12] and veterinary medicine [3, 8–10, 13]. It has been reported that stifle joint distraction facilitates joint access, visualization of intra-articular structures, as well as the diagnosis and treatment required. Besides, it has been described to simplify demanding surgical procedures in human medicine, such as meniscus transplantation [12].

In this study, the preliminary arthroscopic examination of the stifle joint was performed without distraction, but detailed evaluation of intra-articular structures was not always possible in some patients until joint distraction was applied. With distraction, complete access to the menisci and their careful evaluation were possible. The distraction provided by such a device is very steady and progressive, and it has been shown that the amount of distraction can be about 2 mm in the middle of the femoral condyles in a cadaveric study [13], following the mid-condyle method previously described. Though this distance was not consistently measured in this study, it can be assumed that it could be very similar, because the patients had muscle relaxation due to general anaesthesia. Stifle motion is possible during distraction: mild flexion and varus-valgus motion could be accomplished when the surgical procedure required it. The post-procedure radiographic tests performed for the cadaveric study [13] demonstrated that there are no changes in joint stability measured as varus-valgus stability. The clinical experience did not show any specific complication related to the use of the technique. All dogs were managed without bandages post-operatively – only an adhesive tape was applied to the surgical wound when present – and no local effusion, redness or pain were clinically evident. The presence of bone tunnels in the femur and tibia due to the K-wires used for application of the stirrup never showed fissures or cracks predisposing the bone to fracture.

Böttcher et al. [3] previously highlighted the usefulness of distraction in recognizing longitudinal meniscal tears, since a small joint space may make meniscal tear identification complicated. More recently, Winkels et al. [9] stated that some meniscal tears may be misdiagnosed when using external manipulation without instrumented distraction, since external manipulation limits access to the caudal pole of the medial meniscus.

In this study, after diagnosing the meniscal tear, the appropriate treatment was performed while maintaining the distraction. The selection of treatment was based on the diagnosed lesion, since the location of the lesion is important to achieve meniscal healing. With the abaxial third (red zone) being vascularized, the likelihood of meniscal healing is much higher than in the rest of the meniscal parenchyma [4, 20]. For this reason, meniscal suture was performed when the lesion was located in this vascular area.

Although partial meniscectomy could have been accomplished without the distraction manoeuvre, this procedure was performed in a more consistent and safe way due to the space provided by the distraction. The opening of the forceps used for this purpose or the introduction of a radiofrequency probe between joint surfaces is prone to contact with and damage of the joint cartilage. Moreover, radiofrequency ablation could be dangerous if it is performed too close to the joint cartilage [22]. Furthermore, joint evaluation and partial meniscectomy were completed without the need for a fat pad debridement, which may increase morbidity [10].

In the authors’ opinion, meniscal suture could not have been performed without joint distraction. The narrow joint space makes it difficult to move the different instruments around, but using distraction provides safe and easy access to the caudal horn of the medial meniscus.

An important point for the meniscal suture technique is the correct angulation of the spinal needle. It is mandatory to position the scope in the cranio-medial portal and to insert the needle from the cranio-lateral portal, aiming to exit from the caudo-medial area of the stifle. If the needle is inserted in a sagittal direction, like it was if inserted from the cranio-medial portal, it would exit in the popliteal area, making its retrieval impossible.

The only intraoperative complication encountered was the breakage of suture during its passage through the meniscus in one case, but it was not related to the distraction or the amount of joint space. Though the distractor used here was much bulkier compared to other available distractors [3, 10], the force was applied in a more even way because of the bilateral K-wires held by stirrups, while the pin distractor works only on the medial side of the joint. In addition, wire placement in this technique does not interfere with intra-articular structures as is the case for intra-articular distractors. The small size of the wires does not create large bone tunnels, and they can be placed at a distance from the joint line. This is not the case for distractors that use pins, which should be placed quite close to the joint to be effective. A further advantage of this technique is that it can be used in joints other than the stifle [16–18].

The mean time to complete the evaluation and treatment in dogs that received a surgical meniscal treatment (partial meniscectomy and meniscal suture) was approximately double that of the group of dogs that were not treated. The time needed for completing the whole procedure was longer than that reported by Gemmill and colleagues [10], but a comparison cannot be made because the distraction device was different and they did not perform any treatment for the medial meniscus. Moreover, the surgery time is dependent on the skill of the surgeon, and it may be substantially reduced once the surgeon establishes proficiency in performing the procedure [23].

The distraction device used in this study did not cause fractures in a previous cadaveric study with loads of up to 200 N [13]. The clinical postoperative evaluation excluded complications to soft tissues that was not possible to evaluate in the previous cadaveric study.

## Conclusions

Stifle joint distraction is a safe procedure to increase the intra-articular workspace during stifle arthroscopy in dogs. It facilitates joint access and makes possible and easier the consistent visualization of the joint structures and the diagnosis of meniscal pathology. The use of a stifle distractor device should be considered in order to perform partial meniscectomy and meniscal suture, since it facilitates access to the caudal horn of the medial meniscus and therefore suture placement. Although joint distraction is not exempt from complications, no problems related to the distraction procedure have been observed in this case cohort.

## Abbreviations

K-wire: Kirschner wire
